# Cause and Treatment of Facial Neuralgia

**Published:** 1884-05

**Authors:** 


### Cause and Treatment of Facial Neuralgia.

BY MRS. W. M. S., D. D. S.

Neuralgia may be defined as nerve pain. The distinction from any other pain being the fact that in neuralgia the nerve structures themselves are the seat of pain, while in other cases the nerve transmits a knowledge of what exists in surrounding parts. Hence the chief diagnostic point is the absence of heat, redness, swelling, or any external indication of disease.

In facial neuralgia, of course, we deal almost wholly with the fifth pair of cranial nerves. Observations in regard to the actual pathological changes occurring in the nerve structures as a result of neuralgia have been very inadequate. But that changes do occur is beyond question.

Neuralgia may take place as a result of some local irritating cause; or it may originate in the nerve structures; and to distinguish between these conditions is often a diagnostic point of much importance. If a local cause is found and removed, it alone will often be all the treatment required. But even when such cause exists it is not always easy to find. Among the local causes of facial neuralgia may be enumerated the following: Chronic inflammation of the tooth pulp; calcareous formation within the pulp chambers; proliferation of cement about the apex of the root, causing pressure on the nerve entering at that point; periostitis; inflammation of the lining membrane of the antrum, causing pressure on the dental nerves, which often lay in grooves along its walls; teeth crowded, or out of position may cause undue pressure on nerve structures; also, unerupted teeth remaining in the alveolus. These are by no means all of the possible causes, but they are enough to prove the difficulties that may stand in the way of a correct diagnosis. If, however, we are able to ascertain the exciting cause the treatment is evident. But if we are compelled to decide in favor of pure neuralgia or original disease of nerve tissue, other questions at once arise, and first, is it centric or periferic? Is the trouble really at the surface where the patient will usually locate it, or is it farther back on the track of the affected nerve, and only referred to the surface?

Observation seems to show that the most common points of disease are the localities where nerves enter bony canals, as the vicinity of the supraorbital, infra-orbital, and inferior-dental foramina; also, along the course of the branches of the inferior dental nerve, to and into the root of the teeth. Unfortunately the statements of the patient are generally of little value in locating the real seat of pain, there often being no point where pain is continuous.

The treatment of neuralgia should be first palliative and then curative. To allay the present suffering and then to prevent its return. Among palliative measures may be named warm or hot applications, counter irritation, etc. Both aconite and belladonna have been applied with good results. But the most reliable resources are the narcotics and anodynes, and nothing has been so much used as the preparations of opium. Sulphate of morphia may be given; dose, $\frac{1}{6}$ to $\frac{1}{4}$ of a grain. Liquor morphia sulphatis is officinal and contains one grain to the fluid ounce; dose, one to two drachms, equal to $\frac{1}{8}$ to $\frac{1}{4}$ of a grain.

Curative remedies for pure neuralgia must, with our present knowledge of the disease, be prescribed entirely on general principles. Inquiry should be made concerning the performance of all the functions of the body, and measures taken to regulate anything that is abnormal. The restoration of impaired digestion will often effect an entire cure of neuralgia. Tonics are often indicated, than which nothing is better than the preparations of iron.

Practitioners in malarious districts will sometimes meet cases of neuralgia resulting entirely from malarial poisoning, and these without any of the ordinary symptoms of chills, fever, and sweating. In cases of this kind the attacks of neuralgia will be intermittent, but not always with sufficient regularity to prove their malarial origin. But if no sufficient local cause can be found, and the attacks be intermittent, and the patient resides in or has recently visited a malarious district, it is safe to proceed on the supposition that we have the results of malarial poisoning to deal with. The remedies chiefly relied on in these cases are the preparations of cinchona, which possess strong tonic and anti-intermittent properties. Sulphate of quinine is the preparation most used. The dose varies greatly; when used as a febrifuge 16 grains is an ordinary dose, though double this quantity may be given with no ill results. The quinine is best administered between the paroxysms.

The violent neuralgia so often occurring during pregnancy should be met with intelligent and well-directed treatment. The large demand made on the nutritive functions will often leave the general system very insufficiently nourished, and the nervous system does not escape the enfeeblement resulting from this general starvation, so that some slight irritation, that at another time would pass unnoticed, is now sufficient to induce the most severe suffering, and suffering increased by the fact that the whole system is far below its normal tone of resistance.

Of course it is evident that all sources of irritation should be removed as far as circumstances will allow, and then supporting measures resorted to. The nutritive and assimilative functions should be put in their best possible order and the diet ordered with the wisest care, regard being had to the large demand being made on the food supply, and especially for bone building material. Enough calcium should be introduced in some way to meet the new demand without withdrawing the needful supply from the mother. For this purpose let oatmeal constitute a large article of diet. Bread made of unbolted flour should be recommended, and even lime water may be used with the best results, and while, by this means, we may hope to improve the health of the mother, we also provide for good organs of mastication in the child. And just here is a large field where mothers need an education, both for their own benefit and that of their children.

